# A Tale of Tails: Dissecting the Enhancing Effect of Tailed Primers in Real-Time PCR

**DOI:** 10.1371/journal.pone.0164463

**Published:** 2016-10-10

**Authors:** Frank Vandenbussche, Elisabeth Mathijs, David Lefebvre, Kris De Clercq, Steven Van Borm

**Affiliations:** 1 Molecular Platform, Operational Directorate of Viral Diseases, CODA-CERVA, Brussels, Belgium; 2 Vesicular and Exotic Diseases, Operational Directorate of Viral Diseases, CODA-CERVA, Brussels, Belgium; University of Helsinki, FINLAND

## Abstract

Non-specific tail sequences are often added to the 5’-terminus of primers to improve the robustness and overall performance of diagnostic assays. Despite the widespread use of tailed primers, the underlying working mechanism is not well understood. To address this problem, we conducted a detailed *in vitro* and *in silico* analysis of the enhancing effect of primer tailing on 2 well-established foot-and-mouth disease virus (FMDV) RT-qPCR assays using an FMDV reference panel. Tailing of the panFMDV-5UTR primers mainly affected the shape of the amplification curves. Modelling of the raw fluorescence data suggested a reduction of the amplification efficiency due to the accumulation of inhibitors. In depth analysis of PCR products indeed revealed the rapid accumulation of forward-primer derived artefacts. More importantly, tailing of the forward primer delayed artefacts formation and concomitantly restored the sigmoidal shape of the amplification curves. Our analysis also showed that primer tailing can alter utilisation patterns of degenerate primers and increase the number of primer variants that are able to participate in the reaction. The impact of tailed primers was less pronounced in the panFMDV-3D assay with only 5 out of 50 isolates showing a clear shift in Cq values. Sequence analysis of the target region of these 5 isolates revealed several mutations in the inter-primer region that extend an existing hairpin structure immediately downstream of the forward primer binding site. Stabilisation of the forward primer with either a tail sequence or cationic spermine units restored the sensitivity of the assay, which suggests that the enhancing effect in the panFMDV-3D assay is due to a more efficient extension of the forward primer. ur results show that primer tailing can alter amplification through various mechanisms that are determined by both the assay and target region. These findings expand our understanding of primer tailing and should enable a more targeted and efficient use of tailed primers.

## Introduction

Since its conception in the 1980s, the polymerase chain reaction (PCR) has revolutionised many aspects of life sciences. Although PCR was conceived originally as a selective enrichment method [1,2], it was soon adapted to numerous applications and has become an essential tool in any molecular biology laboratory. To facilitate downstream manipulations, non-specific, utility sequences are often added to the 5’-terminus of primers. Some well-known utility sequences include restriction sites for cloning [3,4], universal primer sites for sequencing [5] or RNA polymerase promoter sequences for *in vitro* transcription [6,7]. Tailed primers are also used extensively to improve the robustness and overall performance of diagnostic assays [8–20]. The application of tailed primers in the diagnostic field deserves special attention as it is far more complex than any of the other applications. Instead of incorporating a simple utility sequence, the aim of primer tailing in diagnostics is to improve disease/pathogen detection by enhancing the amplification process. Although numerous tools exist, primer/probe design for highly divergent targets remains challenging due to the lack of universally conserved regions. To minimise the risk of false-negative test results, degenerate or universal bases are often incorporated into primers to capture most of the observed genetic variation. Unfortunately, mismatches between primer and target sequences can have a serious impact on amplification efficiency [21,22]. This problem becomes even more severe when template concentrations are low or inhibitors are present, a situation often encountered when analysing diagnostic samples. Several studies have shown that incorporating tail sequences into primers can increase PCR yield and improve sequencing quality [23–25]. Unfortunately, the underlying mechanisms are not well understood as exemplified by the contradictory results of Armani *et al*. [26], Bessaud *et al*. [9] or Wei and Clover [14] who observed a significant reduction in assay performance when using tailed primers.

In this study, we conducted a detailed analysis of the enhancing effect of tailed primers on 2 well-established foot-and-mouth disease virus (FMDV) diagnostic assays [27,28]. For each assay, several possible modes of action were investigated using a combination of *in vitro* and *in silico* experiments. Using this approach, we were able to demonstrate the existence of various mechanisms with the actual working mechanism being determined by both the assay and target region.

## Materials and Methods

### Assays

Two real-time reverse transcription-polymerase chain reaction (RT-qPCR) assays were used throughout this study. Both assays target highly conserved regions within the FMDV 5’-untranslated region (5UTR) [27] or 3D polymerase gene [28] and are used worldwide for the pan-serotype detection of FMDV. To assess the potential impact of tailed primers, additional primer sets were designed by incorporating A/T rich 5’-tails as described by Afonina *et al*. [23] (S1 Table). All reactions were performed in a LightCycler^®^ 480 Instrument (Roche) using the RNA UltraSense™ One-Step Quantitative RT-PCR System (Thermo Fisher Scientific). Reactions were run in a volume of 20 μl and contained 1x RNA UltraSense™ Reaction Mix, 1 μM forward primer, 1 μM reverse primer, 375 nM 5’-nuclease probe, 1 μl RNA UltraSense™ Enzyme Mix and 7 μL RNA. The cycling conditions consisted of a 30-minute reverse transcription step at 50°C, a 2-minute denaturation step at 95°C and 50 amplification cycles of 10 seconds at 95°C and 1 minute at 60°C. Reactions were performed minimally in triplicate.

The same panFMDV RT-qPCR assays were also run in the presence of the intercalating dye SYBR^®^ Green I using the SuperScript^®^ III Platinum^®^ SYBR^®^ Green One-Step qRT-PCR Kit (Thermo Fisher Scientific). Reactions were performed in a volume of 20 μl containing 1x SYBR^®^ Green Reaction Mix, 1 μM forward primer, 1 μM reverse primer, 1 μl SuperScript^®^ III RT/Platinum^®^ Taq Mix and 5 μl RNA. Cycling conditions were as described above but included an additional melting curve analysis step at the end of the program.

After cycling, quantification cycles (Cq) of each reaction were obtained using the second derivative maximum method of the Lightcycler^®^ 480 software (Roche, release 1.5.0 SP4).

### Viruses and viral RNA

The performance of the assays was tested on an FMDV reference panel (n = 50) containing isolates from all 7 serotypes (S2 Table). The FMDV strains were kindly provided by the World Reference Laboratory for FMDV (The Pirbright Institute, Surrey, UK). All viruses were cultured on swine kidney (SK6) cells, clarified by low-speed centrifugation and stored at -80°C until use.

Viral RNA was extracted from the virus stocks using a NucleoSpin RNA virus kit (Macherey-Nagel) according to the manufacturer's instructions. All RNA dilutions were prepared in 1x TE buffer (10 mM Tris, 0.1 mM EDTA, pH 7.5).

### Synthetic RNA

Similar to other RNA viruses, FMDV isolates are typically composed of a swarm of genetically related variants which are known as viral quasi-species. As these mixed populations could interfere with the high-throughput sequencing (HTS) experiments, a panel of synthetic RNAs was included in the study. Custom gene sequences (306 bp) including both 5UTR and 3D target sequences from a selection of FMDV isolates (n = 5) were constructed and cloned into a plasmid cloning vector (pIDT-Blue) by Integrated DNA Technologies (S3 Table). The entire insert along with an adjacent partial sequence of the cloning vector was amplified using Q5^®^ High-Fidelity DNA Polymerase (New England BioLabs) with primer pair M13F/pr_pIDT-Blue (S1 Table) according to the manufacturer’s instructions. Resulting amplicons were purified with a NucleoSpin Gel and PCR Clean-up kit (Macherey-Nagel) and RNA transcripts were synthesised using a TranscriptAid T7 High Yield Transcription Kit (Thermo Fisher Scientific) as described in the user’s manual. Remaining DNA was removed by treating the RNA twice with Baseline-ZERO^TM^ DNase (Epicentre) and the treated RNA was purified/concentrated using a NucleoSpin RNA Clean-up XS kit (Macherey-Nagel). Finally, the presence of residual template DNA in the RNA transcripts was excluded by qPCR using Platinum^®^ Quantitative PCR SuperMix-UDG (Thermo Fisher Scientific) with the pIDT-Blue primer/probe set (S1 Table) which targets the backbone DNA of the cloning vector.

### Sanger sequencing of primer- and probe binding sites

Complementary DNA was prepared from genomic viral RNA with SuperScript^®^ III Reverse Transcriptase (Thermo Fisher Scientific) in the presence of 2 pmol of each prs_FMDV-3D primer (S1 Table) according to the standard conditions recommended by the supplier. A 453 bp long fragment, containing the entire panFMDV-3D target region as well as part of the upstream and downstream regions, was amplified using Q5^®^ High-Fidelity DNA Polymerase (New England BioLabs) and the pfs_FMDV-3D/prs_FMDV-3D primer pair (S1 Table). All reactions were carried out according to the manufacturer’s instructions and purified with a NucleoSpin Gel and PCR Clean-up kit (Macherey-Nagel).

Sequencing reactions were performed in a volume of 20 μl containing 5 ng purified PCR product, 3.2 pmol of either pfs_FMDV-3D or prs_FMDV-3D primer (S1 Table), 2 μl BigDye^®^ Terminator v3.1 Ready Reaction Mix and 3 μl 5x BigDye^®^ Terminator v3.1 Sequencing Buffer. Cycle-sequencing was performed for 25 cycles following the manufacturer’s recommendations (Applied Biosystems). Unincorporated dye terminators were removed using the BigDye^®^ XTerminator™ Purification kit (Applied Biosystems). Purified sequencing products were analysed on an Applied Biosystems 3130 Genetic Analyzer using 50 cm capillary arrays and POP-7™ polymer. Data were analysed with the Sequencing Analysis Software 5.2.0 (Applied Biosystems).

### High-throughput amplicon sequencing

#### Library preparation and sequencing

*In vitro* synthesised RNA (n = 5) and genomic viral RNA (n = 7) were analysed using the panFMDV-5UTR RT-qPCR assay with both non-tailed and tailed primers as described above but with slight modifications. To assess the impact of PCR artefacts, reactions were performed with either normal dNTPs (5x RNA UltraSense™ Reaction Mix) or hot-start (i.e. CleanAmp™ (TriLink Biotechnologies)) dNTPs (5x standard reaction mix containing 150 mM Tris HCl, 250 mM KCl, 15 mM MgCl_2_ and 2 mM of each hot-start dNTP). Reverse transcription was carried out at 55°C to allow activation of the hot-start dNTPs and the number of cycles was limited to 40. PCR artefacts and amplicons were purified from the RT-qPCR reactions using 1.8 volume of a 30% polyethylene glycol–bead (PEG–bead) solution as described in Clarke *et al*. [29]. TruSeq adapters were ligated to 250 ng of the purified artefacts/amplicons according to the PCR-free workflow of the KAPA Hyper Prep Kit (Kapa Biosystems). Ligation reactions were purified using 0.9 volume of AMPure^®^ XP beads (Beckman Coulter). Resulting libraries were analysed on an Agilent Bioanalyzer DNA 1000 chip (Agilent) and quantified with a KAPA Library Quantification Kit (Kapa Biosystems). Sequencing was performed on a MiSeq benchtop sequencer at the Genomics Core facility of the University Hospitals (KULeuven, Leuven, BE) using a MiSeq Reagent Kit v2 (Illumina) with 2×150 bp paired-end sequencing.

#### Data analysis

Paired-end reads were merged using the fastq_mergepairs command from the USEARCH package [30]. Resulting reads were divided into (nearly complete) amplicons and artefacts based on their size. Artefacts were further subdivided into 4 categories based on the (partial) primer binding sites present on both ends. Data processing was performed within the R statistical environment [31] by combining commands from USEARCH [30] and the Biostrings [32] and Rsamtools [33] packages. To avoid interference from truncated amplicons, all reads containing the complete inter-primer region were removed from the data set using the search_global command. Reads containing 2 forward (‘forward/forward’ artefacts) or reverse (‘reverse/reverse’ artefacts) primer binding sites were identified by running the search_pcr command with either the forward or reverse primer sequences. Similarly, artefacts containing both a forward and reverse primer binding site (‘forward/reverse’ artefacts) were identified in the remaining reads using the search_pcr command with the forward and reverse primer sequences. Contaminating sequences with no resemblance to the FMDV-5UTR amplicons were removed from the remaining reads. All reads that could not be assigned to any of the previous 3 categories were classified as ‘other’ artefacts. Finally, the proportion of each artefact category was calculated for each library (S4 File).

Primer utilisation patterns of the amplicon data set were constructed by counting the number of exact occurrences of each primer variant from the degenerate primer pool using the Tallymer tool [34] from the GenomeTools genome analysis system [35]. To assess the overall impact on primer utilisation, non-tailed and tailed primer counts from all FMDV isolates were combined in a single data set per dNTP type and normalised according to the total count method. Utilisation patterns were explored visually using heat maps to analyse the impact of the target sequence (FMDV isolate), degenerate primer pool composition (relative abundance of each primer variant) and the primer binding affinity (ΔG of each primer variant). Heat maps were generated using the lattice package [36] within the R statistical environment [31] (S4 File). As degenerate bases were synthesised using equal molar concentrations of each base (i.e. standard machine mix procedure), the composition of the resulting degenerate primer pool is expected to be slightly biased due to differences in the coupling efficiency of the various phosphoramidites. The relative abundance of each primer variant was therefore estimated by taking into account the different incorporation rates of each base (T>G>C>A) at the degenerate positions starting from the 3’-end towards the 5’-end of the primer. The binding affinity of each primer variant was calculated by modelling the interaction between each primer variant and its corresponding perfect match target using Visual OMP (DNA Software). Interactions were calculated at a reaction temperature of 60°C with the following reaction conditions: 50 nM monovalent cation, 3.3 nM divalent cation, 32.5 nM of each primer variant and 1 pM of each perfect match target. All other parameters were kept at their default values.

### Modelling of raw fluorescence data

Raw fluorescence data from the LightCycler^®^ 480 Instrument (Roche) was processed within the R statistical environment [31]. Alterations in the shape of the amplification curves were analysed by fitting the mechanistic model described by Carr and Moore (CM3) to the raw fluorescence data of each reaction [37]. As detailed in the supplementary information of the original article, the CM3 model is based on the observation that the amplification efficiency is essentially cycle-dependent due to the accumulation of inhibitors (e.g. double-stranded DNA, pyrophosphate) and, to a lesser extent, depletion of reagents (e.g. primers). Product accumulation is described as a recursive model that depends on 3 variables: the amount of template present after the previous cycle (*prev*), the maximum capacity of the reaction (*max*), and the apparent affinity of accumulated reaction inhibitors (*Kd*). The susceptibility of the reaction to reagent depletion is described by the *max* parameter with lower values indicating stronger dependence on reagent consumption. The *Kd* parameter represents the equilibrium dissociation constant for the enzyme-inhibitor complex and controls the feedback-inhibition term of the model. Although the *Kd* parameter is not directly linked to a particular inhibitor, its value can still be interpreted as the concentration of inhibitor that, at equilibrium, will bind half of the available enzyme in the reaction. Consequently, low *Kd* values imply that amplification is subject to strong inhibition. Model parameters *max* and *Kd* were determined by non-linear regression using the pcrfit function from the qpcR package [38]. Resulting parameters from all models were combined into a single data set using packages dplyr [39] and stringr [40] (S4 File).

### *In silico* analysis

Melting temperatures of non-tailed/tailed panFMDV-3D forward primers were calculated using the nearest-neighbour thermodynamics model as implemented in Visual OMP (DNA Software) [41]. Secondary structure analyses were performed in Visual OMP (DNA Software) by modelling the interactions between the panFMDV-3D forward primer, 5’-nuclease probe and target sequences. Interactions were calculated at a reaction temperature of 60°C with the following reaction conditions: 50 nM monovalent cation, 3.3 nM divalent cation, 1 μM of primer and 1 pM of target. All other parameters were kept at their default values.

## Results

### Impact of tailed primers on the detection of an FMDV reference panel using the panFMDV-5UTR and panFMDV-3D RT-qPCR assays

A reference panel containing 50 FMDV isolates was tested with both non-tailed and tailed primer sets. Analysis of the amplification curves indicated that the use of tailed primers affected the amplification of both the panFMDV-5UTR and panFMDV-3D RT-qPCR assays (S1 File).

**Fig 1:**
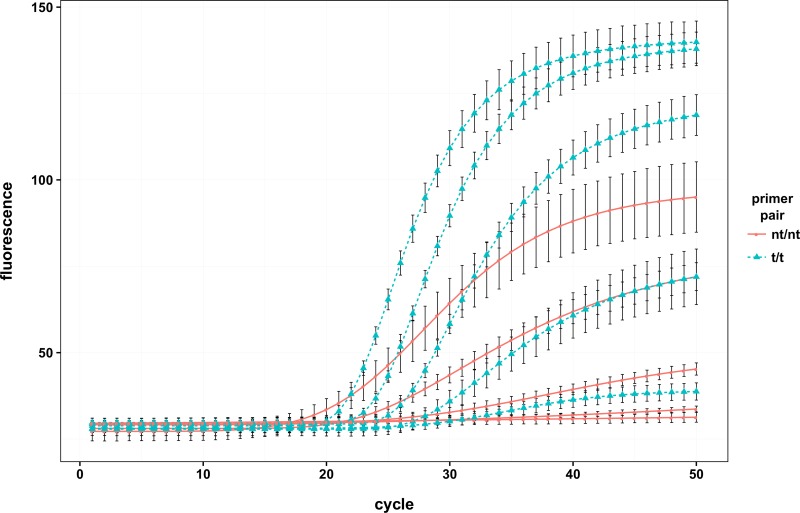
Impact of primer tailing on the panFMDV-5UTR RT-qPCR assay. A 5-fold dilution series of viral genomic RNA of isolate SAT2/ZIM/3/97 was tested in triplicate with the panFMDV-5UTR RT-qPCR assay using either non-tailed or tailed primers. Non-linear regression models were fitted to the raw fluorescence data of each replicate and the resulting models were amalgamated into a single replicate model using the replist function from the qpcR package [38] (S4 File). The figure shows the replicate model of 5 dilutions with error bars representing 1 standard deviation (nt: non-tailed, t: tailed).

Interestingly, both assays responded differently to the use of tailed primers. The most pronounced effect was seen in the panFMDV-5UTR RT-qPCR assay with fluorescence accumulating faster (higher slope) and to higher levels (higher plateau level) in reactions containing tailed primers (Fig 1, worksheet 4 of S1 File). Although the enhancing effect was observed for all serotypes, isolates belonging to the SAT serotypes were generally more affected by primer tailing than any of the other serotypes (S1 Fig). Analysis of an RNA dilution series indicated that the observed differences become even more pronounced at lower target concentrations. In the most affected isolates, amplification curves of the non-tailed primer reactions rapidly lost their sigmoidal shape as the target concentration was reduced. In contrast, amplification curves of the tailed primer reactions remained sigmoidal throughout most of the dilution series thereby increasing the sensitivity of the assay significantly (Fig 1). Modelling of the raw fluorescence data with the CM3 model revealed that the altered shape of the amplification curves is mainly due to differences in the *Kd* parameter which was consistently lower in the non-tailed primer reactions (worksheet 5 of S1 File).

The use of tailed primers had only a moderate effect on the overall shape of the amplification curves of the panFMDV-3D RT-qPCR assay (S2 Fig). These results are also reflected in the modelling data which showed only subtle changes in the model parameters of non-tailed versus tailed primer reactions (S1 File). However, in contrast to the panFMDV-5UTR RT-qPCR assay, 5 isolates showed markedly lower Cq values when tailed primers were used (Table 1).

**Table 1:** Effect of primer tailing on Cq values of the panFMDV-3D RT-qPCR assay. Viral genomic RNA was tested with the panFMDV-3D RT-qPCR assay using either non-tailed or tailed primers. Five isolates of the FMDV reference panel (n = 50) showed markedly lower Cq values when both primers were tailed. The table lists the difference (average ± standard deviation) between reactions containing non-tailed or tailed primers of the 5 aberrant isolates.

isolate	non-tailed–tailed (Cq value)
Asia 1/CAM/2/91	4.51 ± 0.12
C/PHI/11/89	3.79 ± 0.05
O/PHI/7/75	3.98 ± 0.11
O/TUR/2/92	3.04 ± 0.23
SAT2/ZIM/19/89	1.83 ± 0.04

### Impact of tailed primers on the formation of PCR artefacts in the panFMDV-5UTR RT-qPCR assay

The progressive flattening of the amplification curves and markedly lower *Kd* values of the non-tailed reactions suggest that amplification is inhibited more strongly in the non-tailed primer reactions than in the tailed primer reactions. As double-stranded DNA has been shown to be a potent inhibitor of Taq DNA polymerase [42,43], a series of experiments were conducted to study the formation of PCR artefacts in the panFMDV-5UTR RT-qPCR assay. Real-time PCR analysis in the presence of the intercalating dye SYBR^®^ Green I indeed confirmed the presence of non-specific products. A series of reactions using mixed non-tailed/tailed primers were performed to further explore the impact of tailed primers on artefacts formation (S3 Fig). PCR artefacts appeared nearly instantaneously (around cycle 10) in the no-template control when none of the primers contained a 5’-tail (non-tailed/non-tailed reaction). Tailing of the reverse primer (non-tailed/tailed reaction) delayed the formation of artefacts only slightly by approximately 3 cycles. The most pronounced effect was observed when only the forward primer was tailed (tailed/non-tailed reaction), which resulted in a shift of approximately 15 cycles. Interestingly, no additive effect was observed when forward and reverse tailed primers were combined in the same reaction (tailed/tailed reaction). In fact, artefacts appeared approximately 2 cycles earlier in the no-template control of the tailed/tailed reaction compared to the tailed/non-tailed reaction. Melting curve analysis of the amplification products of an FMDV RNA dilution series revealed a clear correlation between the accumulation of PCR artefacts and the extent of PCR inhibition. Whereas nearly no artefacts were present in the more concentrated samples, the amount of non-specific products gradually increased along the dilution series (data not shown). In the most diluted samples, the non-specific products had outcompeted the virus-specific products completely, making these samples indistinguishable from the no-template control (S3 Fig). Although PCR artefacts were present in both non-tailed and tailed reactions, the use of tailed primers delayed their formation significantly. However, once artefacts were formed, they quickly accumulated and reached similar levels than those observed in the non-tailed primer reactions.

**Fig 2:**
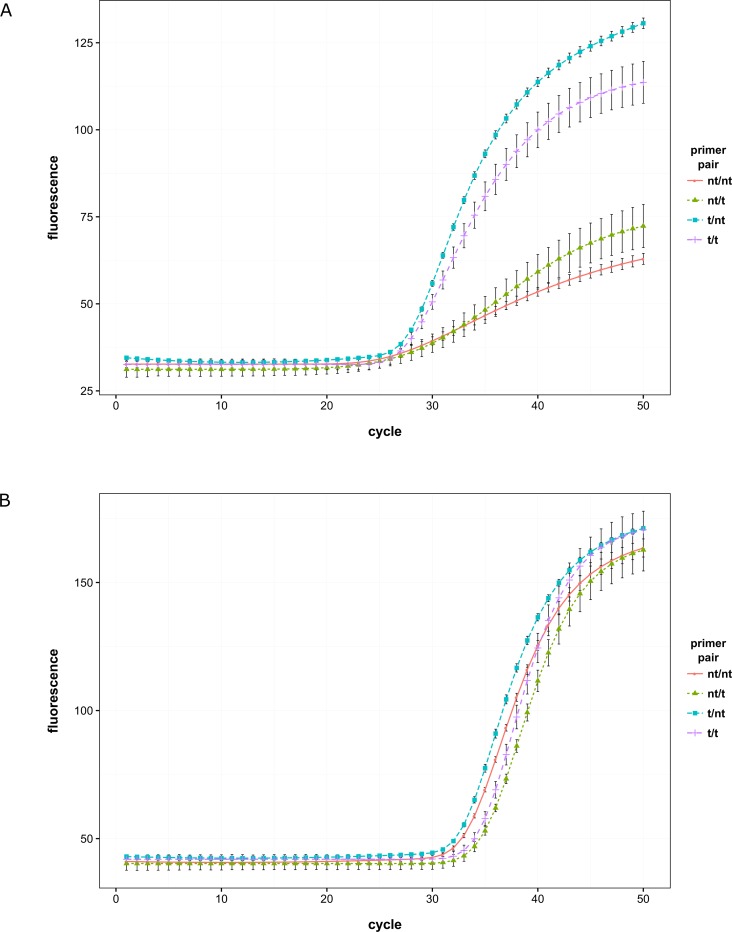
Impact of primer tailing on the panFMDV-5UTR RT-qPCR assay using normal or hot-start dNTPs. Viral genomic RNA of FMDV isolate SAT3/MAL/3/76 was tested with the panFMDV-5UTR RT-qPCR assay using different primer combinations in the presence of either normal dNTPs (panel A) or hot-start dNTPs (panel B). Non-linear regression models were fitted to the raw fluorescence data of each replicate and the resulting models were amalgamated into a single replicate model using the replist function from the qpcR package [38] (S4 File). The figures show the replicate model of each primer combination with error bars representing 1 standard deviation (nt: non-tailed, t: tailed).

The experiments with mixed non-tailed/tailed primer reactions were also performed using the probe based assay format (Fig 2A). The strongest enhancing effect was observed in the tailed/non-tailed primer reactions, which is in agreement with the SYBR^®^ Green I experiments. Although differences in Cq values were negligible, the amplification curves of the tailed/non-tailed reactions were steeper and reached higher fluorescence levels. Tailing of the reverse primer improved the amplification only slightly.

### Impact of PCR artefacts on the performance of the panFMDV-5UTR RT-qPCR assay

To further evaluate the importance of the observed PCR artefacts, the panFMDV-5UTR RT-qPCR assay was carried out in the presence of hot-start nucleotide analogues which contain a thermolabile 3′-tetrahydrofuranyl protecting group [44]. Since 3'-protected dNTPs cannot be incorporated by Taq DNA polymerase, they do not support primer extension/elongation during the lower non-stringent temperatures of reaction setup and can be used as an alternative means to reduce off-target artefact accumulation during PCR. As expected, the use of hot-start dNTPs reduced the formation of PCR artefacts significantly (S4 Fig). However, due to the inefficient activation of the hot-start dNTPs in the reverse transcription step, amplification curves were all shifted to the right by approximately 5 to 6 cycles (Fig 2B). The reduction in PCR artefacts was accompanied by alterations in the shape of the amplification curves similar to the earlier observed tailed primer effect. Although the amplification curves of the non-tailed and tailed primer reactions were nearly indistinguishable from each other, fluorescence accumulation was still somewhat faster in the reactions containing tailed primers (Fig 2B). Nevertheless, sigmoidal amplification curves were visible along the entire dilution series in both the non-tailed and tailed primer reactions (data not shown).

### Impact of tailed primers on PCR artefacts composition in the panFMDV-5UTR RT-qPCR assay

To elucidate the mechanism by which primer tailing reduces PCR artefacts formation, artefacts from reactions containing normal and hot-start dNTPs were analysed by high-throughput sequencing. Classification of the artefacts based on their flanking primer sequences revealed that the composition of PCR artefacts is determined by both the primer and dNTP type. The non-tailed/normal dNTP reactions mainly contained artefacts belonging to the forward/forward (40.60%) and forward/reverse (38.72%) categories (Table 2). Reverse/reverse artefacts were observed only in a few samples and represented less than 0.1% of all artefacts. The vast majority of the forward/forward artefact sequences appeared to be composed entirely of primer sequences whereas all forward/reverse artefact sequences contained additional nucleotides between both primer binding sites. In contrast, most PCR artefacts from the tailed/normal dNTP reactions belonged to the forward/reverse type (87.55%). Although forward/forward artefacts were observed in all samples, they accounted for only 1.92% of all artefacts. Once again, reverse/reverse artefacts were nearly absent.

**Table 2:** Distribution of the different categories of artefacts found in the panFMDV-5UTR PCR reactions using normal or hot-start dNTPs. RNA samples from different FMDV isolates (n = 12) were amplified with non-tailed or tailed primers using either normal or hot-start dNTPs. RT-qPCR products were analysed by high-throughput sequencing to study the contribution of each primer to the formation of PCR artefacts. Artefacts were classified into 4 categories based on the primer sequences present on both ends (FWD: forward, REV: reverse). The table lists the mean proportion and 95% confidence interval of each artefact category.

primer/dNTP	mean (%)			
	(95% confidence interval)			
	FWD/FWD	REV/REV	FWD/REV	OTHER
non-tailed/normal	40.60	0.01	38.72	20.67
	(35.92–45.28)	(0.00–0.03)	(35.15–42.28)	(15.70–25.64)
tailed/normal	1.92	0.01	87.55	10.52
	(1.55–2.30)	(0.00–0.01)	(84.75–90.35)	(7.77–13.26)
non-tailed/hot-start	21.34	0.18	43.94	34.55
	(14.94–27.74)	(0.00–0.41)	(33.07–54.82)	(26.82–42.27)
tailed/hot-start	2.56	0.27	36.21	60.96
	(1.12–3.36)	(0.09–0.45)	(20.25–52.17)	(45.41–76.51)

Analysis of the hot-start dNTP reactions showed a marked reduction of forward/forward artefacts in the non-tailed primer reactions (Table 2). At the same time, slight increases were observed for all other artefact categories. The tailed primer reactions responded differently to the use of hot-start dNTPs. In contrast to the non-tailed primer reactions, a clear shift from the forward/reverse artefacts to the other artefacts category was observed.

### Impact of tailed primers on primer utilisation patterns in the panFMDV-5UTR RT-qPCR assay

**Fig 3:**
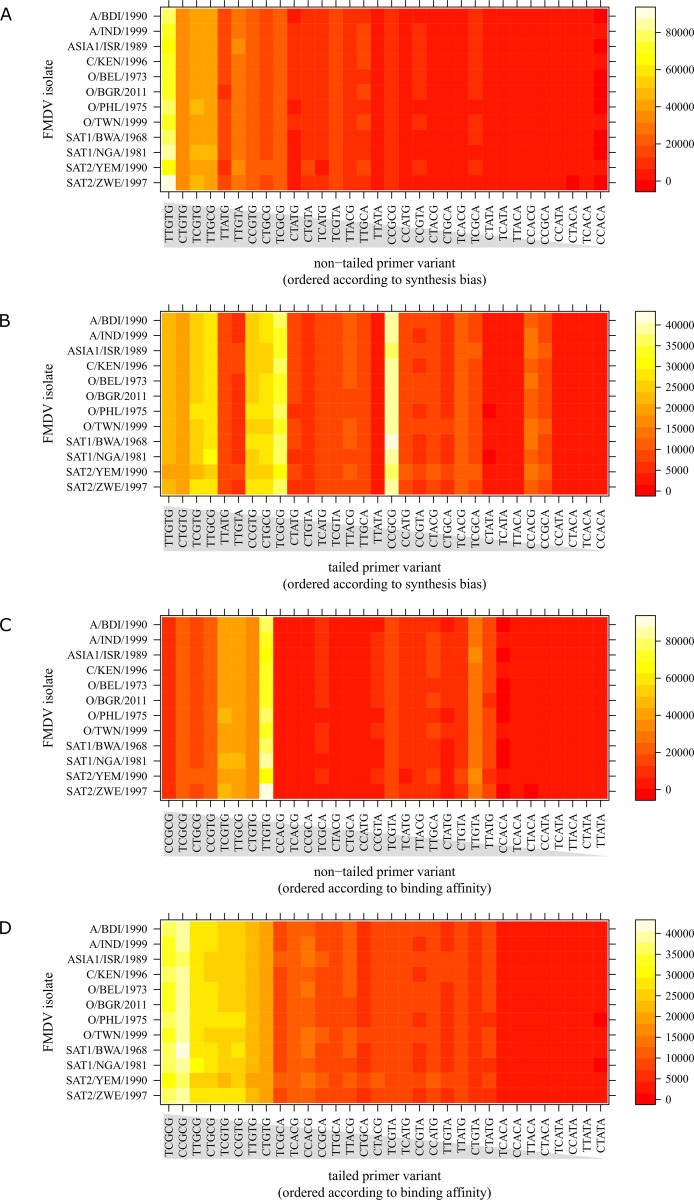
Primer utilisation patterns of the FMDV-5UTR forward primer using hot-start dNTPs. Heat map of forward primer utilisation patterns using non-tailed (A and C) or tailed (B and D) primers. The heat map indicates the abundance of each primer variant for each FMDV isolate, with primers ordered by decreasing synthesis bias (A and B) or decreasing binding affinity (C and D). Primer variants are named according to the nucleotide bases present at the degenerate positions (e.g. primer variant TTGTG corresponds to the primer 5’- CACTTTAAGGTGACATTGGTACTGGTAC -3’).

As the panFMDV-5UTR primers contain multiple degenerate bases, each PCR reaction contains a complex primer pool consisting of 32 forward primers and 8 reverse primers. To assess the impact of tailing on the actual primer utilisation, PCR products of 12 isolates were analysed by high-throughput sequencing. Despite differences in their primer binding sites, highly similar primer utilisation patterns were observed for all isolates (Fig 3). As the PCR reactions were sampled near the plateau phase, the perfect match primer variants did no longer dominate any of the reactions. Although the type of dNTPs used in the PCR reactions did not alter the overall utilisation patterns, the effects of primer tailing were more pronounced in the hot-start dNTPs reactions. Utilisation patterns of both the forward and reverse primers were more uniform in the tailed primer reactions than in the non-tailed primer reactions. Rearranging the forward primer variants according to primer synthesis bias or primer binding affinity revealed that the non-tailed data set is skewed strongly towards the more abundant primer variants (Fig 3A). Utilisation patterns of all non-tailed reactions were dominated by the most abundant TTGTG primer variant, regardless of the FMDV isolate that was used. In contrast, the utilisation patterns of tailed primers were determined mainly by the binding affinity of the primer variants (Fig 3D).

### Sanger sequencing of primer/probe binding regions of the panFMDV-3D RT-qPCR assay

**Fig 4:**
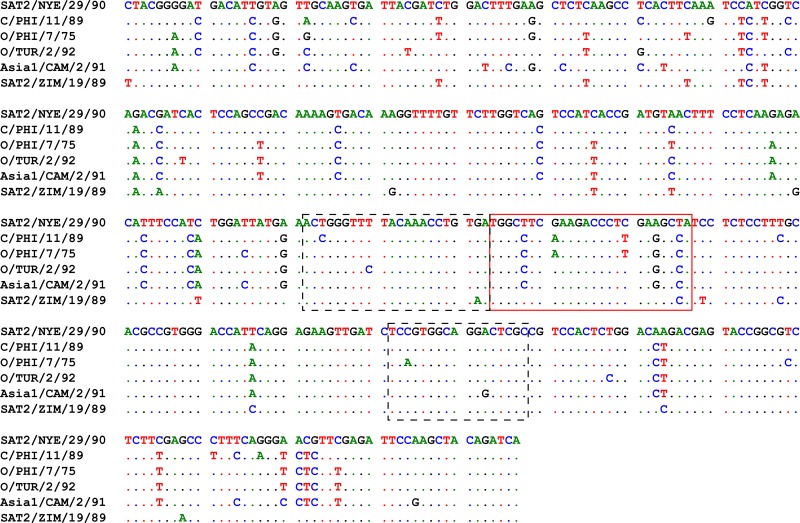
Sequence alignment of a 367 bp fragment of the 3D gene from the control isolate (row 1) and the 5 aberrant isolates (rows 2–6). The region contains the entire panFMDV-3D target region as well as part of the upstream and downstream regions. Primer binding sites are shown in black, dashed boxes whereas the region involved in the hairpin formation is shown in the red box.

**Fig 5:**
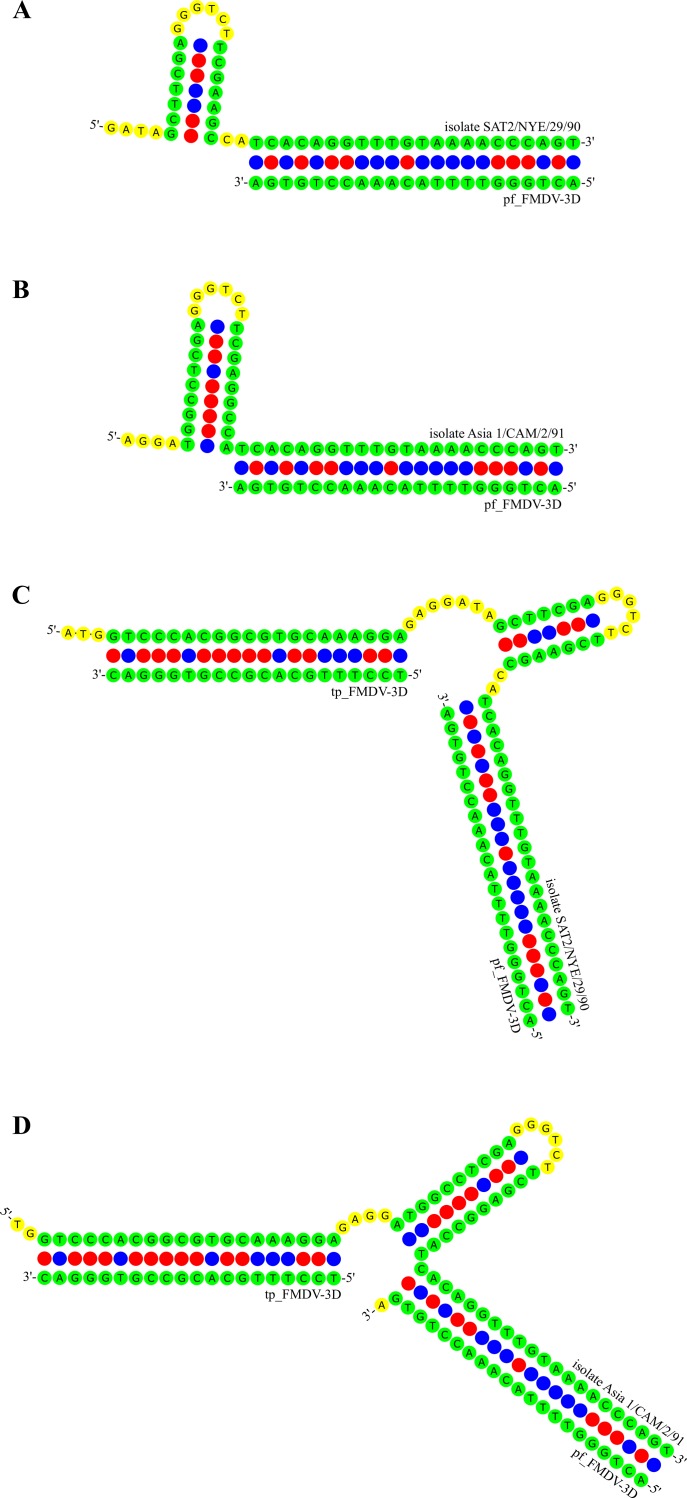
Primer/target and primer/probe/target interactions as predicted by Visual OMP. Interactions between the panFMDV-3D forward primer/probe and target regions from FMDV isolates SAT2/NYE/29/90 (A, C) and Asia 1/CAM/2/91 (B, D). Figures A and B show the predicted interactions between the primer pf_FMDV-3D and the target DNA. Figures C and D show the same interactions in the presence of the probe tp_FMDV-3D.

The fact that the shift in Cq values in the panFMDV-3D RT-qPCR assay was observed only for a limited number of isolates, suggests that it is being caused by nucleotide substitutions in the 3D target region. To test this hypothesis, the entire target regions of the 5 aberrant isolates and 1 control isolate were determined by Sanger sequencing (GenBank accession numbers: KX356531 to KX356536). Although several substitutions were observed in the primer binding sites, none of the isolates contained substitutions in both binding sites or multiple substitutions in a single binding site. Moreover, most of the observed substitutions were predicted to have only a limited impact on amplification (S2 File). However, a comparison of the nucleotide sequences surrounding the primer binding sites revealed a clear substitution pattern downstream of the forward primer binding site (Fig 4). Modelling of the primer/target interactions indicated that these substitutions are not random but extend an existing palindromic sequence. As a consequence, a stable hairpin is formed immediately downstream of the forward primer in all 5 aberrant isolates (Fig 5B and 5D). A similar, albeit shorter, hairpin can be found in the control isolate but this time, the stem of the hairpin and 3’-terminus of the primer binding site are separated by 2 nucleotides (Fig 5A and 5C). No such patterns were observed in the upstream region of the forward primer binding site or the upstream/downstream regions of the reverse primer binding site.

### Impact of target secondary structure on the performance of the panFMDV-3D RT-qPCR assay

**Fig 6:**
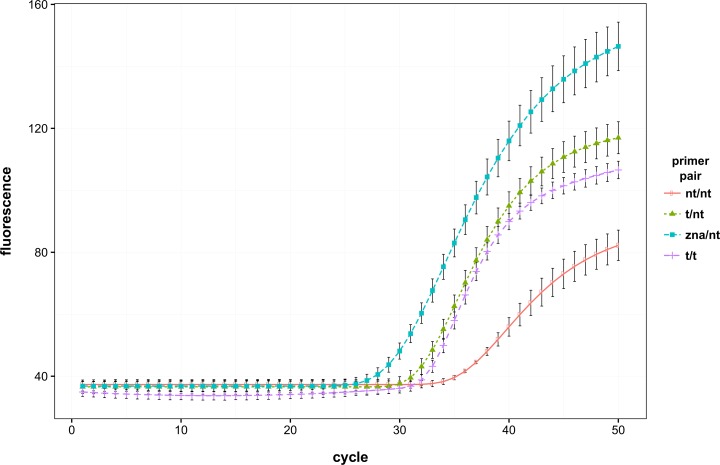
Effect of stabilisation of the forward primer on the performance of the panFMDV-3D RT-qPCR assay. FMDV isolate O/TUR/2/92 was tested with the panFMDV-3D RT-qPCR assay using different primer combinations. Non-linear regression models were fitted to the raw fluorescence data of each replicate and the resulting models were amalgamated into a single replicate model using the replist function from the qpcR package [38] (S4 File). The figure shows the replicate model of each primer combination with error bars representing 1 standard deviation. (nt: non-tailed, t: tailed, zna: zipped nucleic acid).

A series of experiments using mixed non-tailed/tailed primer reactions were conducted to assess the impact of the predicted hairpin structure on the panFMDV-3D RT-qPCR assay’s performance (Fig 6). Analysis of viral genomic RNA of the 5 aberrant FMDV isolates indicated that incorporation of a tail sequence into the forward primer is sufficient to restore the sensitivity of the assay. Interestingly, reactions containing only a tailed forward primer (tailed/non-tailed reactions) performed slightly better than those containing tailed forward and reverse primers (tailed/tailed reactions). Although differences in Cq values were negligible, fluorescence dropped more rapidly in the tailed/tailed reactions. To evaluate the importance of the tail sequence, the same samples were also tested using a non-tailed forward primer that was conjugated with 4 cationic spermine units (zipped nucleic acid/non-tailed reactions). Markedly better results were obtained for nearly all FMDV isolates, both in terms of Cq value and fluorescence accumulation. In contrast to the other reactions, amplification curves of the zipped nucleic acid/non-tailed reactions remained sigmoidal and showed almost no drop in fluorescence (Fig 6).

## Discussion

Despite the widespread use of tailed primers, the exact underlying mechanisms have not yet been elucidated. To better understand the enhancing effect, a detailed analysis was performed on 2 panFMDV RT-qPCR assays. As described previously for other assays [23], the incorporation of tail sequences into the primers altered the amplification of both RT-qPCR assays and improved their robustness and overall performance. Testing of an FMDV reference panel indicated that the enhancing effect depends on both the isolate and target RNA concentration. Interestingly, both assays responded differently to the use of tailed primers which suggests the existence of different working mechanisms. Tailing of the panFMDV-5UTR primers mainly affected the shape of the amplification curves but had little impact on the corresponding Cq values. Modelling of the raw fluorescence data suggested that the altered shape of the non-tailed reactions is due to substantial accumulation of inhibitors (lower *Kd* values). A similar effect on the shape of the amplification curves was observed by Afonina *et al*. in their Varicella-zoster virus real-time PCR (qPCR) assay [23]. One possible explanation for this inhibitory effect is the formation of PCR artefacts [45]. As double-stranded DNA is a potent inhibitor of DNA polymerases [42,46], the accumulation of PCR artefacts is expected to reduce amplification efficiency and hinder detection of challenging samples (low target concentration, mismatches in primer binding sites). Real-time PCR analysis in the presence of an intercalating dye indeed revealed the formation of non-specific products in the early stages of the panFMDV-5UTR RT-qPCR assay. Experiments using mixed non-tailed/tailed primer reactions indicated that the forward primer contributes most to the generation of PCR artefacts. More importantly, tailing of the forward primer was found to delay the formation of artefacts significantly. These results were confirmed by high-throughput sequencing analysis, which showed that forward primer homodimers are the most abundant artefact type in the non-tailed/non-tailed primer reactions but nearly absent in the tailed/tailed primer reactions. A closer examination of the forward primer sequence revealed the presence of a 4 bp palindromic sequence (GTAC) at the 3’-end of the primer. More importantly, the same sequence occurs twice in some of the primer variants (CACYTYAAGRTGACAYTGRTACTGGTAC), which could explain why the forward primer is so prone to homodimer formation. The selective suppression of primer dimers has been described earlier and is due to the inherent structure of homodimers [47]. By definition, all homodimers contain inverted terminal repeats due to the incorporation of the same primer at both ends. As a consequence, homodimers can fold into ‘pan-handle’ like structures which greatly hinders primer binding in consecutive cycles. The extent of this so-called suppression effect depends on several factors including fragment length, GC content of the inverted terminal repeats and primer concentration [48,49]. As high primer concentrations are used in the panFMDV-5UTR RT-qPCR assay, the equilibrium is shifted towards primer binding in the non-tailed/non-tailed primer reactions. Incorporation of 5’-tails into the primers increases the length of the inverted terminal repeats and promotes refolding of the homodimers into the ‘pan-handle’ like structures. The presence of the forward primer homodimers could also explain why artefacts appear so early in the panFMDV-5UTR RT-qPCR. As the homodimers are generated by direct interactions between 2 forward primers, they do not require the presence of ‘foreign’ DNA and are readily available for amplification at the very beginning of the PCR reaction.

The importance of the observed PCR artefacts was also confirmed by performing the panFMDV-5UTR RT-qPCR assay in the presence of hot-start dNTPs which contain a thermolabile 3′-tetrahydrofuranyl protecting group [44]. As described originally by Koukhareva and Lebedev [44], the use of 3’-protected dNTPs reduced the formation of artefacts significantly. More importantly, the amplification curves of the non-tailed and tailed primer reactions were nearly indistinguishable and remained sigmoidal throughout the entire dilution series. High-throughput sequencing analysis further showed that the use of hot-start dNTPs was associated with a significant reduction in the number of forward/forward artefacts in the non-tailed/non-tailed primer reactions, which emphasises the importance of the forward primer homodimers. Nevertheless, the amount of forward primer homodimers was still markedly higher in the non-tailed/non-tailed primer reactions than in the tailed/tailed primer reactions.

As both the forward and reverse primers of the panFMDV-5UTR RT-qPCR are highly degenerated, the observed enhancing effect could also be due, at least partially, to differences in primer utilisation patterns of non-tailed versus tailed primer reactions. As Green *et al*. pointed out, PCR products are generated by 2 mechanisms: a ‘natural template/primer’ annealing process and an ‘artificial template/primer’ annealing process [50]. Although artificial template/primer interactions can occur only from cycle 3 onwards, they rapidly dominate the reaction as artificial template/primer interactions yield exponential amplification, while natural template/primer annealing interactions yield linear amplification. To identify potential differences in primer utilisation patterns, PCR products of both non-tailed and tailed primer reactions were analysed by high-throughput sequencing. In contrast to Green *et al*., PCR reactions were sampled near the plateau phase to focus our analysis on the artificial template/primer interactions. As a consequence, the primer utilisation patterns of all FMDV isolates were very similar and the perfect match primer variants did no longer dominate the PCR reactions. More interestingly, high-throughput sequencing showed that the utilisation patterns of tailed primer reactions are more uniform. Our results also suggest that primer utilisation in the non-tailed reactions is, at least partially, driven by the composition of the degenerate primer pool, with the most abundant TTGTG primer variant being over-represented in all data sets. As expected, the next most represented group of primer variants are all closely related to the dominant TTGTG primer variant and differ by only a single mismatch. The utilisation patterns of the tailed primer reactions were markedly different and appeared to be shifted towards the strongest binding primer variants. Most likely, the incorporation of a 5’-tail sequence in the initial PCR cycles neutralises the destabilising effect of mismatches in the artificial template/primer annealing complexes that arise when a primer variant interacts with a different artificial template (e.g. primer variant 2 binding to an artificial template containing primer variant 1). As a consequence, more primer variants are expected to be able to participate in the PCR reaction which ultimately leads to the selection of primer variants with the highest binding affinity. This hypothesis was already suggested by Regier and Shi but was never supported with actual data [25].

The impact of tailed primers was rather limited in the panFMDV-3D RT-qPCR assay with only 5 isolates showing a clear shift in Cq values. As no enhancing effect was apparent in any of the other isolates, the study of the panFMDV-3D assay was focused on these 5 aberrant isolates. In contrast to the panFMDV-5UTR assay, only small alterations in the shape of the amplification curves were observed. A similar enhancing effect was described earlier by Afonina *et al*. in their Enterovirus RT-qPCR assay [23]. As the enhancing effect was observed only for a limited number of FMDV isolates, we hypothesized that the reduced sensitivity in these isolates was due to mutations in the primer binding sites. Surprisingly, sequence analysis of the corresponding amplicons revealed a clear mutational pattern downstream of the forward primer binding site but not in the primer binding sites themselves. A systematic analysis of all publicly available FMDV genomes indicates that this pattern is not particularly rare (52 out of 525 isolates) and occurs in nearly all serotypes (S3 File). The observation that these mutations extend an existing hairpin structure immediately downstream of the primer binding site suggests that the reduced sensitivity in these isolates is caused by an inefficient extension of the forward primer. *In silico* analysis of the target DNA indeed predicts the formation of a highly stable hairpin structure (Tm > 72.0°C) in the 5 aberrant isolates. As the predicted melting temperature of the forward primer varies between 62.9 and 67.0°C, a substantial amount of the target DNA is likely to be folded into the hairpin structure before the primer can anneal. Incorporation of a 5’-tail sequence into the forward primer is predicted to increase the melting temperature to 72.7°C (artificial template/primer complex) which would allow the primer to be extended already at higher temperatures. This hypothesis is further supported by the observation that stabilisation of the forward primer alone (tailed/non-tailed or zipped nucleic acid/non-tailed) is sufficient to restore the sensitivity of the assay. A similar phenomenon was reported by Liu *et al*. who observed severe inhibition of a conventional PCR targeting exon H of the human factor IX gene which was found to be due to a single mutation downstream of one of the primers [51]. Despite the fact that the authors ruled out secondary structure as a possible cause, modelling of the target region with Visual OMP predicts the formation of a hairpin structure that contains the 3’-end of the primer binding site. Although sequences outside the primer/probe binding sites are often neglected, the results presented in this study demonstrate that small changes in the inter-primer region can severely impair amplification. It is therefore recommended to perform a detailed secondary structure analysis of the entire target region whenever designing or evaluating primer/probe sets.

## Conclusion

In this study, we have shown that primer tailing can alter amplification through various mechanisms with the actual working mechanism being determined by both the assay and target region. Using 2 panFMDV RT-qPCR assays as model systems, we were able to identify 3 mechanisms: (i) suppression of primer artefacts formation, (ii) alteration of primer utilisation patterns and (iii) improved extension through partially folded target regions. This list is not intended to be exhaustive as our study was limited to only 2 assays. Nevertheless, the findings presented in this study can help researchers determine when primer tailing might be considered to improve suboptimal (RT-)PCR assays. Although numerous applications are conceivable, one field that could benefit substantially from the use of primer tailing is viral diagnostics. Due to the high genetic heterogeneity found in RNA viruses, it is not always possible to design optimal primer/probe sets for each virus. As shown in our study, primer tailing can, in some cases, enhance the performance of suboptimal (RT-)PCR assays. However, despite the potential benefits, systematic tailing of primers is not recommended as it does not necessarily yield the best results. Although some of the encountered problems can be resolved more effectively using hot-start dNTPs or spermine-conjugated primers, the use of tailed primers offers an attractive alternative as it yields similar results at a much lower cost. Finally, this study highlights the importance of good test design and the need for periodic evaluation of existing diagnostic assays.

## Supporting Information

S1 File Raw fluorescence data and model parameters. An FMDV reference panel (n = 50) was tested in triplicate with the panFMDV-5UTR and panFMDV-3D RT-qPCR assays using both non-tailed and tailed primer sets (sheets 1–3). The raw fluorescence data of each reaction were modelled using a nonlinear sigmoidal model (sheet 4) or the CM3 model (sheet 5).
(XLSX)

S2 File Impact of the observed substitutions in the pf_FMDV-3D binding site of the 5 aberrant FMDV isolates on the melting temperature of the primer/target heterodimer. Interactions between the panFMDV-3D forward primer and the target sequences from the 5 aberrant FMDV isolates were modelled in Visual OMP (DNA Software) using a reaction temperature of 60°C and the following reaction conditions: 50 nM monovalent cation, 3.3 nM divalent cation, 1 μM of primer and 1 pM of target. All other parameters were kept at their default values.
(XLSX)

S3 File FMDV isolates from GenBank containing the same extended palindromic structure in the panFMDV-3D target region. FMDV-3D sequences were retrieved from GenBank on 16/11/2015 and aligned using MAFFT v7.245 [52]. The resulting sequence alignment was trimmed to the same region shown in Fig 4 to facilitate comparison between the sequences from Fig 4 and S3 File. The file contains all sequences from GenBank which contain the same extended palindromic structure in the panFMDV-3D target region.
(FAS)

S4 File R scripts used in this study for data analysis. (TXT)

S1 Fig Correlation between the FMDV serotype and the enhancing effect of primer tailing in the panFMDV-5UTR RT-qPCR assay. An FMDV reference panel (n = 50) was tested in triplicate with the panFMDV-5UTR RT-qPCR assay using either non-tailed or tailed primer sets. Non-linear regression models were fitted to the raw fluorescence data of each replicate. Resulting models were used to calculate the slope of the amplification curve at the first derivative maximum and the fluorescence at cycle 50. The figures show the average slope (A) and average plateau level (B) of the non-tailed versus tailed PCR reactions.
(TIFF)

S2 Fig Correlation between the FMDV serotype and the enhancing effect of primer tailing in the panFMDV-3D RT-qPCR assay. An FMDV reference panel (n = 50) was tested in triplicate with the panFMDV-3D RT-qPCR assay using either non-tailed or tailed primer sets. Non-linear regression models were fitted to the raw fluorescence data of each replicate. Resulting models were used to calculate the slope of the amplification curve at the first derivative maximum and the fluorescence at cycle 50. The figures show the average slope (A) and average plateau level (B) of the non-tailed versus tailed PCR reactions.
(TIFF)

S3 Fig Impact of primer tailing on the formation of PCR artefacts in the panFMDV-5UTR RT-qPCR assay. Viral genomic RNA of FMDV isolate SAT3/MAL/3/76 and a no-template control were tested in triplicate with different panFMDV-5UTR primer combinations in the presence of the intercalating dye SYBR® Green I. Non-linear regression models were fitted to the raw fluorescence data of each replicate and the resulting models were amalgamated into a single replicate model using the replist function from the qpcR package [38] (S4 File). The figure shows the replicate model of each primer combination with error bars representing 1 standard deviation (nt: non-tailed, t: tailed, NTC: no-template control).
(TIFF)

S4 Fig Impact of primer tailing and hot-start dNTPs on the formation of PCR artefacts in the panFMDV-5UTR RT-qPCR assay. Viral genomic RNA of FMDV isolate SAT3/MAL/3/76 and a no-template control were tested in triplicate with different panFMDV-5UTR primer combinations using either normal (A) or hot-start dNTPs (B). All reactions were carried out in the presence of the intercalating dye SYTO 16. Non-linear regression models were fitted to the raw fluorescence data of each replicate and the resulting models were amalgamated into a single replicate model using the replist function from the qpcR package [38] (S4 File). The figures show the replicate model of each primer combination with error bars representing 1 standard deviation (nt: non-tailed, t: tailed, NTC: no-template control).
(TIFF)

S1 Table Primers and probes used throughout this study. (DOCX)

S2 Table FMDV isolates used throughout this study. (DOCX)

S3 Table FMDV isolates used to construct the custom gene sequences. (DOCX)

## References

[1] Saiki RK, Gelfand DH, Stoffel S, Scharf SJ, Higuchi R, Horn GT, et al Primer-directed enzymatic amplification of DNA with a thermostable DNA polymerase. Science. 1988; 239(4839): 487–491. 10.1126/science.2448875 2448875

[2] Saiki RK, Scharf S, Faloona F, Mullis KB, Horn GT, Erlich HA, et al Enzymatic amplification of beta-globin genomic sequences and restriction site analysis for diagnosis of sickle cell anemia. Science. 1985; 230(4732): 1350–1354. 10.1126/science.2999980 2999980

[3] Scharf SJ, Horn GT, Erlich HA. Direct cloning and sequence analysis of enzymatically amplified genomic sequences. Science. 1986; 233(4768): 1076–1078. 10.1126/science.3461561 3461561

[4] Kaufman DL, Evans GA. Restriction endonuclease cleavage at the termini of PCR products. Biotechniques. 1990; 9(3): 304, 306. 2171587

[5] Daigle D, Simen BB, Pochart P. High-throughput sequencing of PCR products tagged with universal primers using 454 life sciences systems. Curr Protoc Mol Biol. 2011; Chapter 7: Unit7.5. 10.1002/0471142727.mb0705s96 21987058

[6] Van Gelder RN, von Zastrow ME, Yool A, Dement WC, Barchas JD, Eberwine JH. Amplified RNA synthesized from limited quantities of heterogeneous cDNA. Proc Natl Acad Sci U S A. 1990; 87(5): 1663–1667. 10.1073/pnas.87.5.1663 1689846 PMC53542

[7] Baugh L, Hill A, Brown E, Hunter C. Quantitative analysis of mRNA amplification by *in vitro* transcription. Nucleic Acids Res. 2001; 29(5): e29 10.1093/nar/29.5.e29 11222780 PMC29742

[8] Arif M, Aguilar-Moreno GS, Wayadande A, Fletcher J, Ochoa-Corona FM. Primer modification improves rapid and sensitive *in vitro* and field-deployable assays for detection of high plains virus variants. Appl Environ Microbiol. 2014; 80(1): 320–327. 10.1128/AEM.02340-13 24162574 PMC3910988

[9] Bessaud M, Jegouic S, Joffret M-L, Barge C, Balanant J, Gouandjika-Vasilache I, et al Characterization of the genome of human enteroviruses: Design of generic primers for amplification and sequencing of different regions of the viral genome. J Virol Methods. 2008; 149(2): 277–284. 10.1016/j.jviromet.2008.01.027 18329732

[10] Bilodeau GJ, Martin FN, Coffey MD, Blomquist CL. Development of a multiplex assay for genus- and species-specific detection of *Phytophthora* based on differences in mitochondrial gene order. Phytopathology. 2014; 104(7): 733–748. 10.1094/PHYTO-09-13-0263-R 24915428

[11] Haegeman A, Zro K, Vandenbussche F, Demeestere L, Van Campe W, Ennaji MM, et al Development and validation of three Capripoxvirus real-time PCRs for parallel testing. J Virol Methods. 2013; 193(2): 446–451. 10.1016/j.jviromet.2013.07.010 23850698

[12] Hymas WC, Mills A, Ferguson S, Langer J, She RC, Mahoney W, et al Development of a multiplex real-time RT-PCR assay for detection of influenza A, influenza B, RSV and typing of the 2009-H1N1 influenza virus. J Virol Methods. 2010; 167(2): 113–118. 10.1016/j.jviromet.2010.03.020 20362006

[13] Leifer I, Blome S, Beer M, Hoffmann B. Development of a highly sensitive real-time RT-PCR protocol for the detection of Classical swine fever virus independent of the 5′ untranslated region. J Virol Methods. 2011; 171(1): 314–317. 10.1016/j.jviromet.2010.11.014 21111760

[14] Wei T, Clover G. Use of primers with 5′ non-complementary sequences in RT-PCR for the detection of nepovirus subgroups A and B. J Virol Methods. 2008; 153(1): 16–21. 10.1016/j.jviromet.2008.06.020 18639585

[15] Wensman JJ, Jäderlund KH, Gustavsson MH, Hansson-Hamlin H, Karlstam E, Lilliehöök I, et al Markers of Borna disease virus infection in cats with staggering disease. J Feline Med Surg. 2012; 14(8): 573–582. 10.1177/1098612X12446638 22553310 PMC11104187

[16] Zeng M, Xu X, Zhu C, Chen J, Zhu Q, Lin S, et al Clinical and molecular epidemiology of norovirus infection in childhood diarrhea in China. J Med Virol. 2012; 84(1): 145–151. 10.1002/jmv.22248 22028199

[17] Plaskon NE, Adelman ZN, Myles KM. Accurate strand-specific quantification of viral RNA. PLoS One. 2009; 4(10): e7468 10.1371/journal.pone.0007468 19847293 PMC2760750

[18] Price EP, Dale JL, Cook JM, Sarovich DS, Seymour ML, Ginther JL, et al Development and validation of *Burkholderia pseudomallei*-specific real-time PCR assays for clinical, environmental or forensic detection applications. PLoS One. 2012; 7(5): e37723 10.1371/journal.pone.0037723 22624061 PMC3356290

[19] Timmons C, Dobhal S, Fletcher J, Ma LM. Primers with 5’ flaps improve the efficiency and sensitivity of multiplex PCR assays for the detection of *Salmonella* and *Escherichia coli* O157:H7. J Food Prot. 2013; 76(4): 668–673. 10.4315/0362-028X.JFP-12-428 23575131

[20] Waitumbi JN, Gerlach J, Afonina I, Anyona SB, Koros JN, Siangla J, et al Malaria prevalence defined by microscopy, antigen detection, DNA amplification and total nucleic acid amplification in a malaria-endemic region during the peak malaria transmission season. Tropical medicine & international health: TM & IH. 2011; 16(7): 786–793. 10.1111/j.1365-3156.2011.02773.x 21447064

[21] Stadhouders R, Pas SD, Anber J, Voermans J, Mes THM, Schutten M. The effect of primer-template mismatches on the detection and quantification of nucleic acids using the 5′ nuclease assay. The Journal of Molecular Diagnostics. 2010; 12(1): 109–117. 10.2353/jmoldx.2010.090035 19948821 PMC2797725

[22] Lefever S, Pattyn F, Hellemans J, Vandesompele J. Single-nucleotide polymorphisms and other mismatches reduce performance of quantitative PCR assays. Clin Chem. 2013; 59(10): 1470–1480. 10.1373/clinchem.2013.203653 24014836

[23] Afonina I, Ankoudinova I, Mills A, Lokhov S, Huynh P, Mahoney W. Primers with 5’ flaps improve real-time PCR. Biotechniques. 2007; 43(6): 770, 772, 774. 10.2144/000112631 18251253

[24] Binladen J, Gilbert MTP, Campos PF, Willerslev E. 5’-tailed sequencing primers improve sequencing quality of PCR products. Biotechniques. 2007; 42(2): 174, 176. 10.2144/000112316 17373481

[25] Regier JC, Shi D. Increased yield of PCR product from degenerate primers with nondegenerate, nonhomologous 5’ tails. Biotechniques. 2005; 38(1): 34, 36, 38. 10.2144/05381bm02 15679081

[26] Armani A, Giusti A, Guardone L, Castigliego L, Gianfaldoni D, Guidi A. Universal primers used for species identification of foodstuff of animal origin: Effects of oligonucleotide tails on PCR amplification and sequencing performance. Food Anal Methods. 2015; 1–11. 10.1007/s12161-015-0301-9

[27] Reid SM, Ferris NP, Hutchings GH, Zhang Z, Belsham GJ, Alexandersen S. Detection of all seven serotypes of foot-and-mouth disease virus by real-time, fluorogenic reverse transcription polymerase chain reaction assay. J Virol Methods. 2002; 105(1): 67–80. 10.1016/s0166-0934(02)00081-2 12176143

[28] Callahan JD, Brown F, Osorio FA, Sur JH, Kramer E, Long GW, et al Use of a portable real-time reverse transcriptase-polymerase chain reaction assay for rapid detection of foot-and-mouth disease virus. J Am Vet Med Assoc. 2002; 220(11): 1636–1642. 10.2460/javma.2002.220.1636 12051502

[29] Clarke AC, Prost S, Stanton J-AL, White WTJ, Kaplan ME, Matisoo-Smith EA. From cheek swabs to consensus sequences: an A to Z protocol for high-throughput DNA sequencing of complete human mitochondrial genomes. BMC genomics. 2014; 15: 68 10.1186/1471-2164-15-68 24460871 PMC3922791

[30] Edgar RC. Search and clustering orders of magnitude faster than BLAST. Bioinformatics. 2010; 26(19): 2460–2461. 10.1093/bioinformatics/btq461 20709691

[31] R Core Team. R: A language and environment for statistical computing [Internet]. 2015. Available: https://www.R-project.org/.

[32] Pages H, Aboyoun P, Gentleman R, DebRoy S. Biostrings: String objects representing biological sequences, and matching algorithms [Internet]. Available: https://www.bioconductor.org/packages/release/bioc/html/Biostrings.html.

[33] Morgan M, Pagès H, Obenchain V, Hayden N. Rsamtools: Binary alignment (BAM), FASTA, variant call (BCF), and tabix file import [Internet]. Available: http://bioconductor.org/packages/release/bioc/html/Rsamtools.html.

[34] Kurtz S, Narechania A, Stein JC, Ware D. A new method to compute K-mer frequencies and its application to annotate large repetitive plant genomes. BMC Genomics. 2008; 9(1): 1–18. 10.1186/1471-2164-9-517 18976482 PMC2613927

[35] Gremme G, Steinbiss S, Kurtz S. GenomeTools: A comprehensive software library for efficient processing of structured genome annotations. IEEE/ACM Trans Comput Biol Bioinform. 2013; 10(3): 645–656. 10.1109/TCBB.2013.68 24091398

[36] Sarkar D. Lattice: Multivariate data visualization with R [Internet]. Springer; 2008 Available: http://lmdvr.r-forge.r-project.org.

[37] Carr AC, Moore SD. Robust quantification of polymerase chain reactions using global fitting. PLoS One. 2012; 7(5): e37640 10.1371/journal.pone.0037640 22701526 PMC3365123

[38] Spiess A-N, Feig C, Ritz C. Highly accurate sigmoidal fitting of real-time PCR data by introducing a parameter for asymmetry. BMC Bioinformatics. 2008; 9: 221 10.1186/1471-2105-9-221 18445269 PMC2386824

[39] Wickham H, Francois R. dplyr: A grammar of data manipulation [Internet]. 2015. Available: https://CRAN.R-project.org/package=dplyr.

[40] Wickham H. stringr: Simple, consistent wrappers for common string operations [Internet]. 2015. Available: https://CRAN.R-project.org/package=stringr.

[41] SantaLucia JJ, Hicks D. The thermodynamics of DNA structural motifs. Annu Rev Biophys Biomol Struct. 2004; 33: 415–440. 10.1146/annurev.biophys.32.110601.141800 15139820

[42] Kainz P, Schmiedlechner A, Strack HB. Specificity-enhanced hot-start PCR: addition of double-stranded DNA fragments adapted to the annealing temperature. Biotechniques. 2000; 28(2): 278–282. 10683737 10.2144/00282st04

[43] SantaLucia JJ. Physical principles and Visual-OMP software for optimal PCR design. Methods Mol Biol. 2007; 402: 3–34. 10.1007/978-1-59745-528-2_1 17951788

[44] Koukhareva I, Lebedev A. 3′-protected 2′-deoxynucleoside 5′-triphosphates as a tool for heat-triggered activation of polymerase chain reaction. Anal Chem. 2009; 81(12): 4955–4962. 10.1021/ac8026977 19438248 PMC2712722

[45] Brownie J, Shawcross S, Theaker J, Whitcombe D, Ferrie R, Newton C, et al The elimination of primer-dimer accumulation in PCR. Nucleic Acids Res. 1997; 25(16): 3235–3241. 10.1093/nar/25.16.3235 9241236 PMC146890

[46] Kainz P. The PCR plateau phase–towards an understanding of its limitations. Biochimica et Biophysica Acta (BBA)-Gene Structure and Expression. 2000; 1494(1): 23–27. 10.1016/s0167-4781(00)00200-1 11072065

[47] Lukyanov KA, Launer GA, Tarabykin VS, Zaraisky AG, Lukyanov SA. Inverted terminal repeats permit the average length of amplified DNA fragments to be regulated during preparation of cDNA libraries by polymerase chain reaction. Anal Biochem. 1995; 229(2): 198–202. 10.1006/abio.1995.1402 7485972

[48] Dai Z-M, Zhu X-J, Chen Q, Yang W-J. PCR-suppression effect: Kinetic analysis and application to representative or long-molecule biased PCR-based amplification of complex samples. J Biotechnol. 2007; 128(3): 435–443. 10.1016/j.jbiotec.2006.10.018 17194496

[49] Shagin DA, Lukyanov KA, Vagner LL, Matz MV. Regulation of average length of complex PCR product. Nucleic Acids Res. 1999; 27(18): e23 10.1093/nar/27.18.e23 10471753 PMC148615

[50] Green SJ, Venkatramanan R, Naqib A. Deconstructing the polymerase chain reaction: Understanding and correcting bias associated with primer degeneracies and primer-template mismatches. PLoS One. 2015; 10(5): e0128122 10.1371/journal.pone.0128122 25996930 PMC4440812

[51] Liu Q, Thorland EC, Sommer SS. Inhibition of PCR amplification by a point mutation downstream of a primer. Biotechniques. 1997; 22(2): 292–4. 9043701 10.2144/97222st01

[52] Katoh K, Standley DM. MAFFT multiple sequence alignment software version 7: Improvements in performance and usability. Mol Biol Evol. 2013; 30(4): 772–780. 10.1093/molbev/mst010 23329690 PMC3603318

