# Supplementary figures and images for: A Tale of Tails: Dissecting the Enhancing Effect of Tailed Primers in Real-Time PCR
Source: PLoS One. 2016 Oct 10;11(10):e0164463. doi: 10.1371/journal.pone.0164463 (PMC5056738; doi:10.1371/journal.pone.0164463)

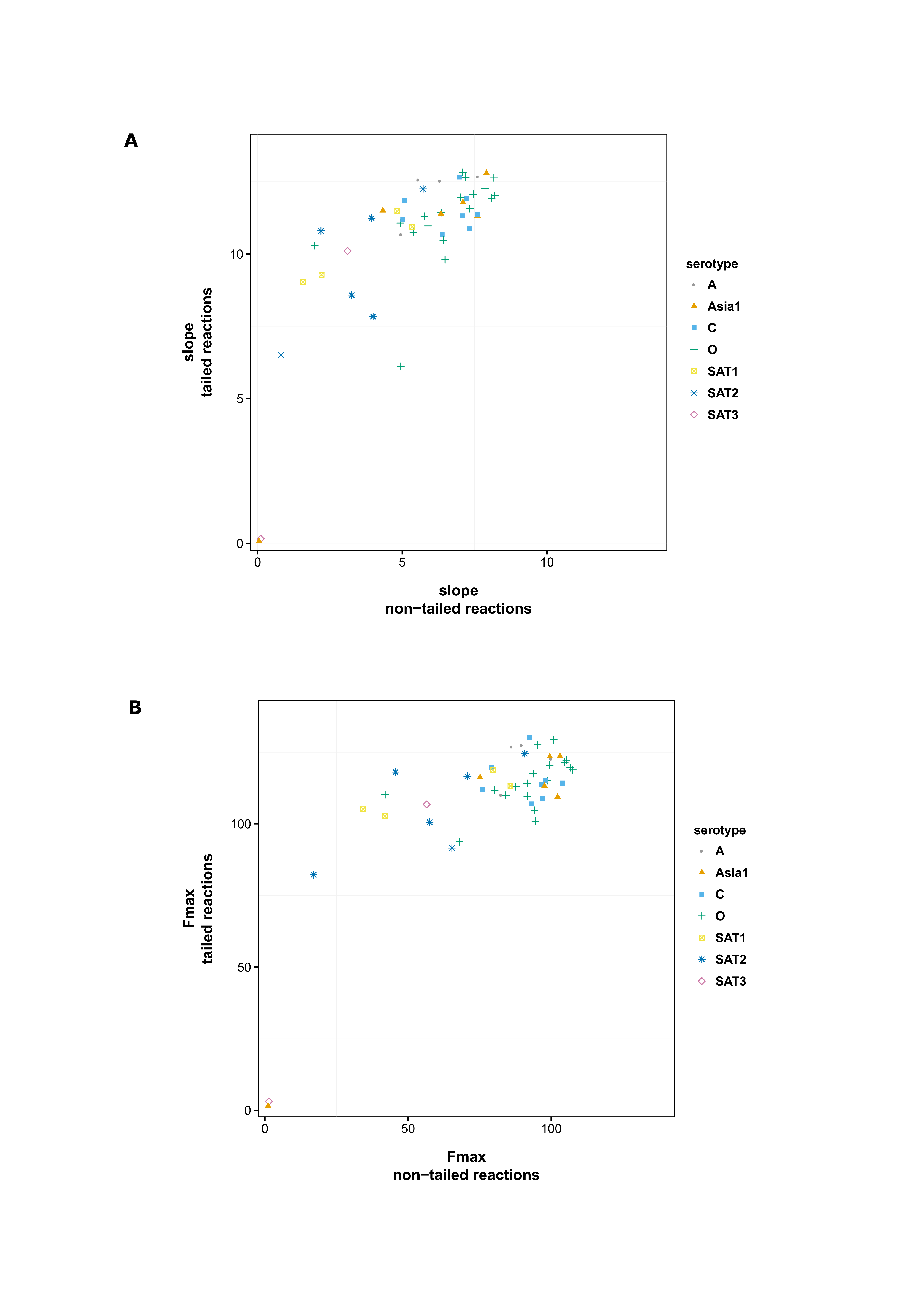

Supplement: S1 Fig — An FMDV reference panel (n = 50) was tested in triplicate with the panFMDV-5UTR RT-qPCR assay using either non-tailed or tailed primer sets. Non-linear regression models were fitted to the raw fluorescence data of each replicate. Resulting models were used to calculate the slope of the amplification curve at the first derivative maximum and the fluorescence at cycle 50. The figures show the average slope (A) and average plateau level (B) of the non-tailed versus tailed PCR reactions. (TIFF) [file pone.0164463.s005.tiff]

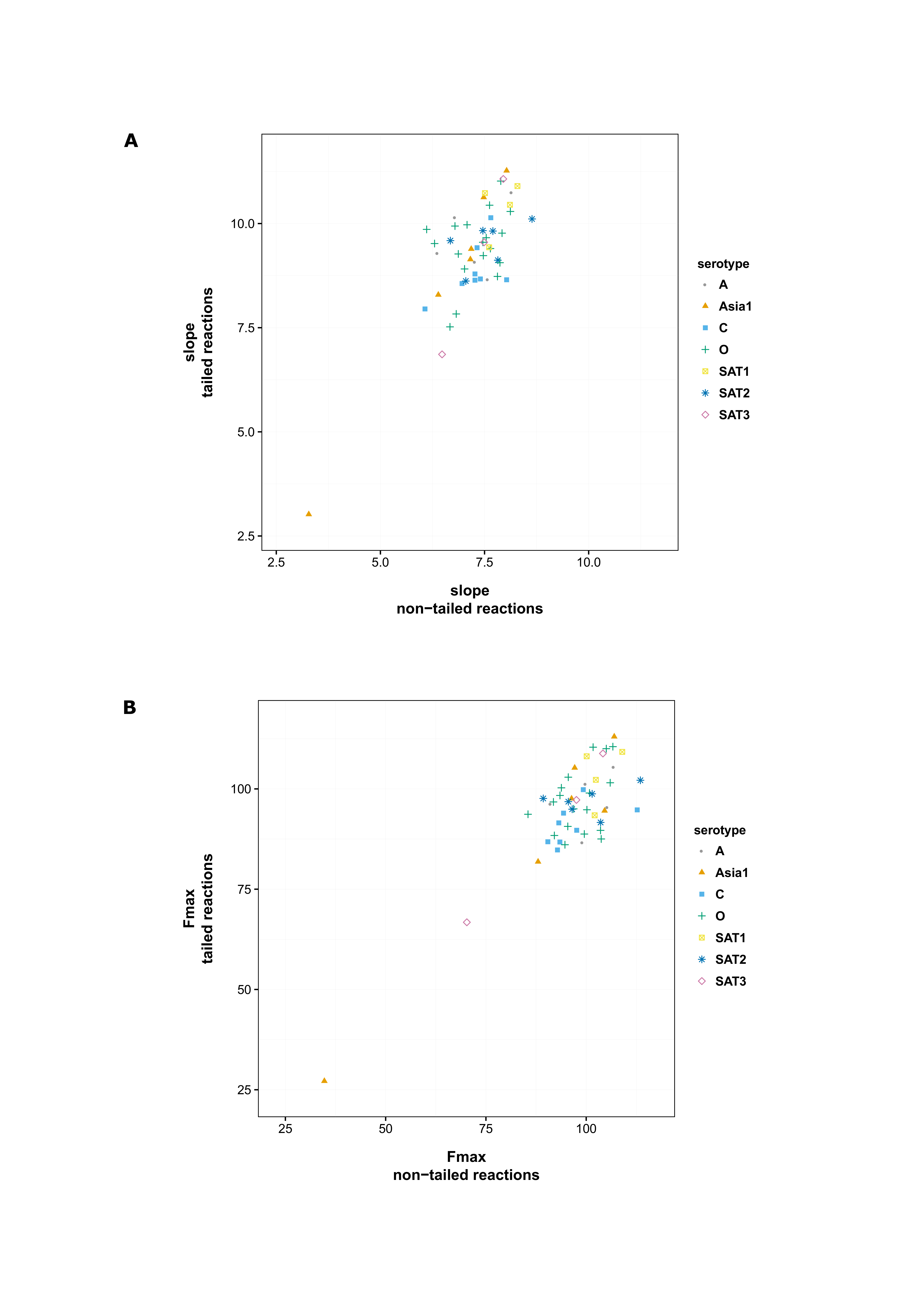

Supplement: S2 Fig — An FMDV reference panel (n = 50) was tested in triplicate with the panFMDV-3D RT-qPCR assay using either non-tailed or tailed primer sets. Non-linear regression models were fitted to the raw fluorescence data of each replicate. Resulting models were used to calculate the slope of the amplification curve at the first derivative maximum and the fluorescence at cycle 50. The figures show the average slope (A) and average plateau level (B) of the non-tailed versus tailed PCR reactions. (TIFF) [file pone.0164463.s006.tiff]

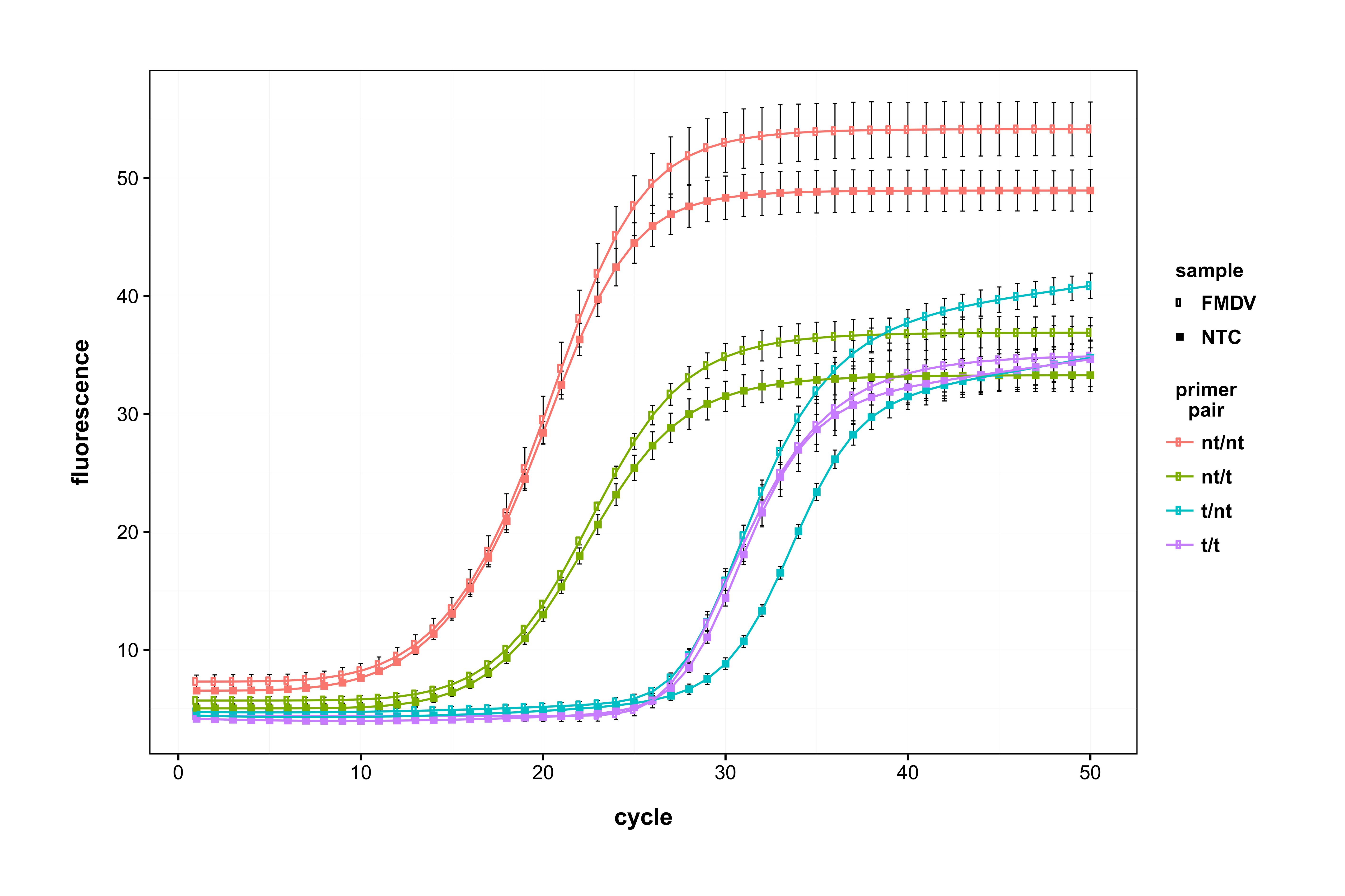

Supplement: S3 Fig — Viral genomic RNA of FMDV isolate SAT3/MAL/3/76 and a no-template control were tested in triplicate with different panFMDV-5UTR primer combinations in the presence of the intercalating dye SYBR® Green I. Non-linear regression models were fitted to the raw fluorescence data of each replicate and the resulting models were amalgamated into a single replicate model using the replist function from the qpcR package [38] (S4 File). The figure shows the replicate model of each primer combination with error bars representing 1 standard deviation (nt: non-tailed, t: tailed, NTC: no-template control). (TIFF) [file pone.0164463.s007.tiff]

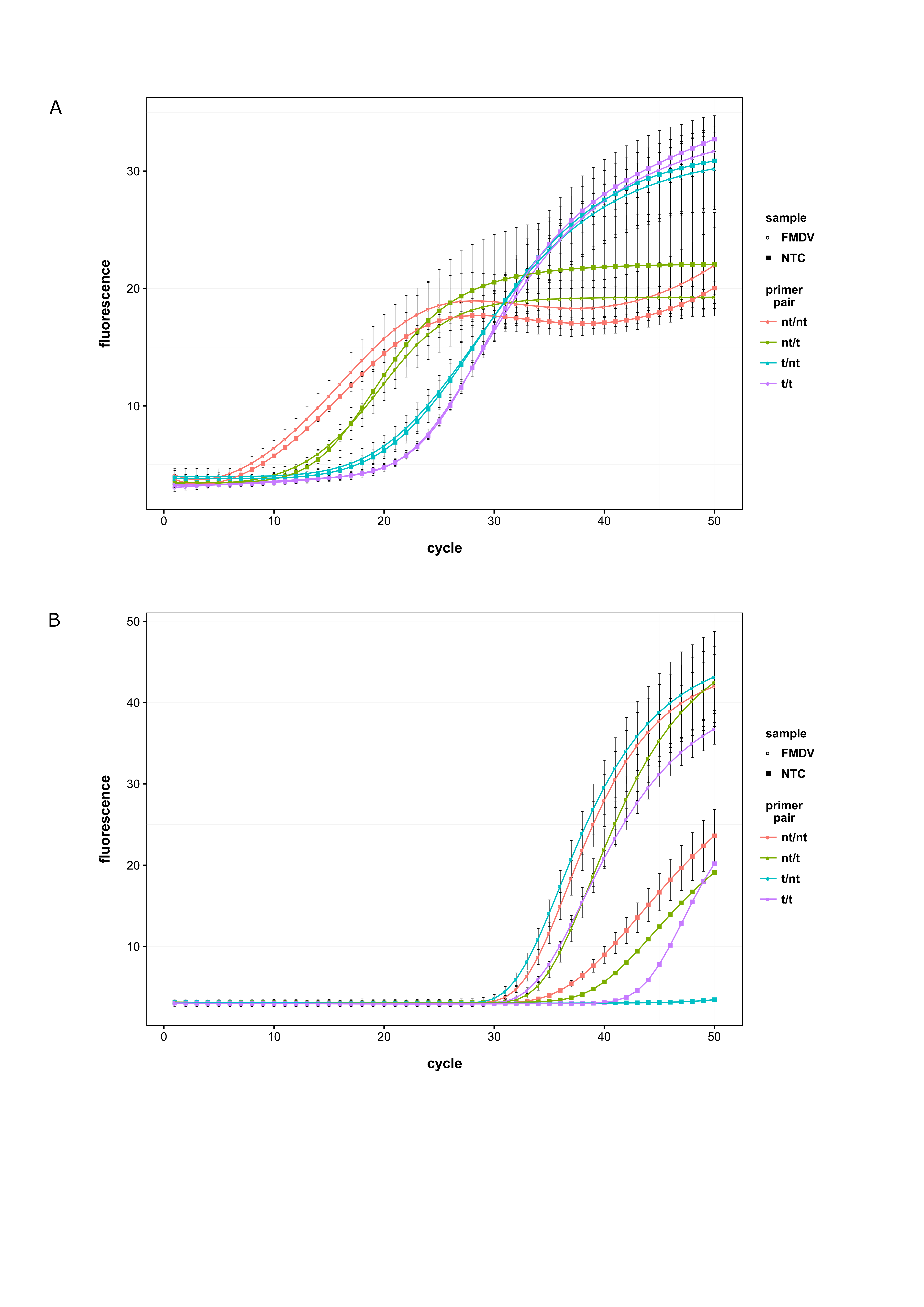

Supplement: S4 Fig — Viral genomic RNA of FMDV isolate SAT3/MAL/3/76 and a no-template control were tested in triplicate with different panFMDV-5UTR primer combinations using either normal (A) or hot-start dNTPs (B). All reactions were carried out in the presence of the intercalating dye SYTO 16. Non-linear regression models were fitted to the raw fluorescence data of each replicate and the resulting models were amalgamated into a single replicate model using the replist function from the qpcR package [38] (S4 File). The figures show the replicate model of each primer combination with error bars representing 1 standard deviation (nt: non-tailed, t: tailed, NTC: no-template control). (TIFF) [file pone.0164463.s008.tiff]
