# Supplementary material for: A Tale of Tails: Dissecting the Enhancing Effect of Tailed Primers in Real-Time PCR
Source: PLoS One. 2016 Oct 10;11(10):e0164463. doi: 10.1371/journal.pone.0164463 (PMC5056738; doi:10.1371/journal.pone.0164463)
Supplement: S1 Table — (DOCX) [file pone.0164463.s009.docx]

**S1 Table. Primers and probes used throughout this study.**

| **primer/probe^a^** | **sequence (5’-3’)^b^** |
| --- | --- |
| pf_FMDV-5UTR/n | CACYTYAAGRTGACAYTGRTACTGGTAC |
| pf_FMDV-5UTR/t | aataaataatctcCACYTYAAGRTGACAYTGRTACTGGTAC |
| pr_FMDV-5UTR/n | CAGATYCCRAGTGWCICITGTTA |
| pr_FMDV-5UTR/t | aataaataatctcCAGATYCCRAGTGWCICITGTTA |
| tp_FMDV-5UTR | FAM/CCTCGGGGTACCTGAAGGGCATCC/BHQ-1 |
| pf_FMDV-3D/n | ACTGGGTTTTACAAACCTGTGA |
| pf_FMDV-3D/t | aataaataatcctACTGGGTTTTACAAACCTGTGA |
| pf_FMDV-3D/zna | ZNA4-ACTGGGTTTTACAAACCTGTGA |
| pr_FMDV-3D/n | GCGAGTCCTGCCACGGA |
| pr_FMDV-3D/t | aataaataatctcGCGAGTCCTGCCACGGA |
| tp_FMDV-3D | FAM/TCCTTTGCACGCCGTGGGAC/BHQ-1 |
| M13F | GTTTTCCCAGTCACGACGTT |
| pf_pIDT-Blue | GCTCCAGCTTTTGTTCCCTTTAG |
| pr_pIDT-Blue | CCCAGGCTTTACACTTTATGCTTC |
| tp_pIDT-Blue | FAM/TAGCTGTTT/ZEN/CCTGTGTGAAATTGTTATCCGCTC/IABkFQ |
| pfs_FMDV-3D/T1-V1 | gttagactgtgactcttgaAGGGRGTTGAGCTGGACACWTAYACCATGATCTC |
| pfs_FMDV-3D/T1-V2 | gttagactgtgactcttgaAGGGWGTCGAGCTGTCGCACTACACCATGATTTC |
| prs_FMDV-3D/T2-V1 | aggatttaccagacactatagGTTCACCCAACGCAGGTARAG |
| prs_FMDV-3D/T2-V2 | aggatttaccagacactatagGTTCACCCAWCGCAGGTAAAG |

^a^ pf: forward primer, pr: reverse primer, tp: 5’-nuclease probe, pfs: forward sequencing primer, prs: reverse sequencing primer

^b^ 5’-tail sequences are shown in lowercase
