# Supplementary material for: A Tale of Tails: Dissecting the Enhancing Effect of Tailed Primers in Real-Time PCR
Source: PLoS One. 2016 Oct 10;11(10):e0164463. doi: 10.1371/journal.pone.0164463 (PMC5056738; doi:10.1371/journal.pone.0164463)
Supplement: S2 Table — (DOCX) [file pone.0164463.s010.docx]

**S2 Table. FMDV isolates used throughout this study.**

| **isolate** | **serotype** | **use^a^** |
| --- | --- | --- |
| A/BUN/4/90 | A | RT-qPCR, HTS |
| A/IRN/11/96 | A | RT-qPCR |
| A/SEN/10/97 | A | RT-qPCR |
| A5/FRA/1/68 | A | RT-qPCR |
| A22/IRQ/24/64 | A | RT-qPCR |
| Asia 1/BAN/1/79 | Asia 1 | RT-qPCR |
| Asia 1/CAM/9/80 | Asia 1 | RT-qPCR |
| Asia 1/CAM/2/91 | Asia 1 | RT-qPCR |
| Asia 1/MAY/34/95 | Asia 1 | RT-qPCR |
| Asia 1/OMN/2/82 | Asia 1 | RT-qPCR |
| Asia 1/Shamir/ISR/89 | Asia 1 | RT-qPCR, HTS |
| C/BAN/2/92 | C | RT-qPCR |
| C/BHU/2/91 | C | RT-qPCR |
| C/KEN/5/96 | C | RT-qPCR, HTS |
| C/NEP/1/94 | C | RT-qPCR |
| C/PHI/11/89 | C | RT-qPCR |
| C/SAU/1/84 | C | RT-qPCR |
| C/SRL/1/84 | C | RT-qPCR |
| C1/Noville/SWI/65 | C | RT-qPCR |
| O/ETH/3/90 | O | RT-qPCR |
| O/HKN/15/90 | O | RT-qPCR |
| O/HKN/1/92 | O | RT-qPCR |
| O/HKN/24/96 | O | RT-qPCR |
| O/KUW/1/96 | O | RT-qPCR |
| O/MAY/17/96 | O | RT-qPCR |
| O/PHI/7/75 | O | RT-qPCR, HTS |
| O/SAU/11/85 | O | RT-qPCR |
| O/SAU/16/91 | O | RT-qPCR |
| O/SAU/12/94 | O | RT-qPCR |
| O/SAU/5/95 | O | RT-qPCR |
| O/SAU/8/95 | O | RT-qPCR |
| O/TUR/5/90 | O | RT-qPCR |
| O/TUR/7/91 | O | RT-qPCR |
| O/TUR/2/92 | O | RT-qPCR |
| O/TUR/11/96 | O | RT-qPCR |
| O/UKG/11/2001 | O | RT-qPCR |
| O1/MAN/TUR/69 | O | RT-qPCR |
| SAT1/BOT/1/68 | SAT1 | RT-qPCR |
| SAT1/NIG/10/81 | SAT1 | RT-qPCR, HTS |
| SAT1/ZIM/25/89 | SAT1 | RT-qPCR |
| SAT1/UGA/47/71 | SAT1 | RT-qPCR |
| SAT2/NYE/29/90 | SAT2 | RT-qPCR, HTS |
| SAT2/ZIM/9/89 | SAT2 | RT-qPCR |
| SAT2/ZIM/19/89 | SAT2 | RT-qPCR |
| SAT2/ZIM/11/91 | SAT2 | RT-qPCR |
| SAT2/ZIM/1/97 | SAT2 | RT-qPCR |
| SAT2/ZIM/3/97 | SAT2 | RT-qPCR, HTS |
| SAT3/MAL/3/76 | SAT3 | RT-qPCR |
| SAT3/UGA/92/70 | SAT3 | RT-qPCR |
| SAT3/ZIM/4/99 | SAT3 | RT-qPCR |

^a^ HTS: high-throughput sequencing, RT-qPCR: real-time reverse transcription-polymerase chain reaction
