# Supplementary material for: A Tale of Tails: Dissecting the Enhancing Effect of Tailed Primers in Real-Time PCR
Source: PLoS One. 2016 Oct 10;11(10):e0164463. doi: 10.1371/journal.pone.0164463 (PMC5056738; doi:10.1371/journal.pone.0164463)
Supplement: S3 Table — (DOCX) [file pone.0164463.s011.docx]

**S3 Table. FMDV isolates used to construct the custom gene sequences.**

| **isolate** | **serotype** | **use^a^** | **accession number** |
| --- | --- | --- | --- |
| IND258/99 | A | HTS | HM854023 |
| O1 Brugge | O | HTS | AY593817 |
| BUL/32/2011 | O | HTS | JX040490 |
| O Penghu/99 | O | HTS | AY593833 |
| SAT1 BOT 1/68 | SAT1 | HTS | AY593845 |

^a^ HTS: high-throughput sequencing
